# Complete Genome Sequence of *Xanthomonas arboricola* pv. juglandis Strain DW3F3, Isolated from a *Juglans regia* L. Bacterial Blighted Fruitlet

**DOI:** 10.1128/genomeA.00023-18

**Published:** 2018-02-22

**Authors:** Benzhong Fu, Qianqian Chen, Mi Wei, Jieqian Zhu, Lulu Zou, Guoyuan Li, Lihua Wang

**Affiliations:** a College of Life Science and Technology, Hubei Engineering University, Xiaogan, China; b Hubei Key Laboratory of Quality Control of Characteristic Fruits and Vegetables, Xiaogan, China; c The College of Natural Resources and Environment, South China Agricultural University, Guangzhou, China

## Abstract

Here, we report the complete genome sequence of *Xanthomonas arboricola* pv. juglandis DW3F3, a strong pathogenic strain isolated from blighted walnut immature fruit (*Juglans regia* L. cv. Qingxiang). The genome consists of a single chromosome (5,144 kb).

## GENOME ANNOUNCEMENT

The Gram-negative gammaproteobacterium *Xanthomonas arboricola* pv. juglandis, the causal agent of walnut bacterial blight, is the most important bacterial disease of the Persian (English) walnut (*Juglans regia* L.) in China and many other countries around the world (1–4). The disease can occur in a walnut nursery or orchard and is considered a major cause of quality decrease and yield loss. The pathogen attacks buds, catkins, leaves, and young twigs, with the most serious infection occurring on immature fruits. Symptoms begin as small dark-brown spots with a yellowish halo that develop into larger areas of dark, varnished, and eventually dead tissue. Most of the field production loss due to walnut bacterial blight is caused by young-fruit infection directly, which can lead to their premature drop and decline in nut quality of fruit that remain on the tree (5). Although the cultivars resistant to the pathogen are different, all commercially grown walnut varieties are considered susceptible to the pathogen (6, 7).

China has become the country with the greatest amount of walnut planting and production; during 2016, the harvest area was greater than 487,007 ha, and the total production was more than 1.78 million tons, with the quantity and area both being more than 50% of global production (http://www.fao.org/faostat/en/#data/QC). Here, we describe the genome sequence of *X. arboricola* pv. juglandis strain DW3F3, a strong pathogenic strain isolated from a blighted cv. Qingxiang (which is one of the major cultivars in Hubei Province) fruit in Danjiangkou, Hubei, China, in May 2015.

The genome sequence was obtained using the MiSeq system (Illumina, Inc., San Diego, CA) with two paired-end libraries, which generated 1,861,577,316 and 2,013,450,108 reads. The estimated genome coverage with these two libraries was ∼72-fold. After sequencing, reads were assembled using the SPAdes software (8), resulting in 8 contigs, with an *N*_50_ value of 1,706,766 bp and a smallest contig of 1,696 bp. These contigs were ordered using the CONTIGuator software (9) against many genomes of the same genus. The genome of *X. campestris* strain 17 (GenBank accession number CP011256) was used as a reference due to better synteny and number of contigs mapped. The initial scaffold was later subjected to a finishing process using the CLC Genomics Workbench software, and gaps were removed with recursive rounds of short reads mapped against the scaffold with the GapCloser and GapFiller tools (10). The annotation step was performed using the NCBI Prokaryotic Genome Annotation Pipeline.

The final genome had eight large contigs without gaps, and the total size was 5,144,431 nucleotides with 4,441 putative open reading frames. The G+C content was 65.36%, and there were 7 rRNA genes and 53 tRNA genes. Further comparison analysis of the genome with different strains in the same pathovar and other pathovars is now under way. These data will allow us to identify specific secreted pathogenic effectors that might explain the pathogenicity, pathogen copper resistance genes, and the interactions between the pathogen and the host.

### Accession number(s).

The *Xanthomonas arboricola* pv. juglandis strain DW3F3 genome sequence and annotation data have been deposited at DDBJ/EMBL/GenBank under the accession number PNRC00000000.

## References

[1] Du Plessis HJ, van der Westhuizen TJ 1995 Identification of *Xanthomonas campestris* pv. juglandis from (Persian) English walnut nursery trees in South Africa. J Phytopathol 143:449–454. doi:10.1111/j.1439-0434.1995.tb04552.x.

[2] Scortichini M, Marchesi U, Di Prospero P 2001 Genetic diversity of *Xanthomonas arboricola* pv. juglandis (synonyms: *X. campestris* pv. juglandis; *X*. juglandis pv. juglandis) strains from different geographical areas shown by repetitive polymerase chain reaction genomic fingerprinting. J Phytopathol 149:325–332. doi:10.1046/j.1439-0434.2001.00628.x.

[3] Kałuzna M, Pulawska J, Waleron M, Sobiczewski P 2014 The genetic characterization of *Xanthomonas arboricola* pv. juglandis, the causal agent of walnut blight in Poland. Plant Pathol 63:1404–1416. doi:10.1111/ppa.12211.

[4] Giovanardi D, Bonneau S, Gironde S, Fischer-Le Saux M, Manceau C, Stefani E 2016 Morphological and genotypic features of *Xanthomonas arboricola* pv. juglandis populations from walnut groves in Romagna region, Italy. Eur J Plant Pathol 145:1–16. doi:10.1007/s10658-015-0809-2.

[5] Scotton M, Bortolin E, Fiorin A, Belisario A 2015 Environmental and pathogenic factors inducing brown apical necrosis on fruit of English (Persian) walnut. Phytopathology 105:1427–1436. doi:10.1094/PHYTO-01-15-0029-R. 26214123

[6] Woeste KE, McGranahan GH, Schroth MN 1992 Variation among Persian walnuts in response to inoculation with *Xanthomonas campestris* pv. juglandis. J Am Soc Hort Sci 117:527–531.

[7] Frutos D, López G 2012 Search for *Juglans regia* genotypes resistant/tolerant to *Xanthomonas arboricola* pv. juglandis in the framework of cost action 873. J Plant Pathol 94:S1.37–S1.46.

[8] Nurk S, Bankevich A, Antipov D, Gurevich A, Korobeynikov A, Lapidus A, Prjibelsky A, Pyshkin A, Sirotkin A, Sirotkin Y, Stepanauskas R, McLean J, Lasken R, Clingenpeel SR, Woyke T, Tesler G, Alekseyev MA, Pevzner PA 2013 Assembling genomes and mini-metagenomes from highly chimeric reads. *In* Deng M, Jiang R, Sun F, Zhang X (ed), Research in computational molecular biology. RECOMB 2013. Lecture notes in computer science, vol 7821 Springer, Berlin, Germany.

[9] Galardini M, Biondi EG, Bazzicalupo M, Mengoni A 2011 CONTIGuator: a bacterial genomes finishing tool for structural insights on draft genomes. Source Code Biol Med 6:11. doi:10.1186/1751-0473-6-11. 21693004 PMC3133546

[10] Boetzer M, Pirovano W 2012 Toward almost closed genomes with GapFiller. Genome Biol 13:R56. doi:10.1186/gb-2012-13-6-r56. 22731987 PMC3446322

